# Spread of the Invasive Mosquitoes *Aedes aegypti* and *Aedes albopictus* in the Black Sea Region Increases Risk of Chikungunya, Dengue, and Zika Outbreaks in Europe

**DOI:** 10.1371/journal.pntd.0004664

**Published:** 2016-04-26

**Authors:** Muhammet M. Akiner, Berna Demirci, Giorgi Babuadze, Vincent Robert, Francis Schaffner

**Affiliations:** 1 Recep Tayyip Erdogan University, Faculty of Arts and Sciences, Department of Biology, Fener, Rize, Turkey; 2 Kafkas University, Molecular Biology and Genetics Department, Kars, Turkey; 3 Division of Virology and Molecular Biology, National Centre for Disease Control and Public Health (NCDC), Tbilisi, Georgia; 4 Research Unit MIVEGEC, Institut de Recherche pour le Développement, Montpellier, France; 5 Avia-GIS, Zoersel, Belgium; Centers for Disease Control and Prevention, Puerto Rico, UNITED STATES

The yellow fever and dengue mosquito *Aedes aegypti* previously flourished around the Mediterranean and Black Sea for decades until the 1950s, and was responsible of large outbreaks of both yellow fever and dengue [1]. The first well-described large dengue outbreak in Greece in 1927–28 caused more than 1 million cases (90% of the population in Athens) with 1000–1500 fatalities. The disappearance of *Ae*. *aegypti* from the European continent in Mediterranean, Black Sea, and Macaronesian biogeographical regions [2] is not well understood and its return in these regions raises concerns about a possible resurgence of the pathogens that can be transmitted by this vector species. Besides, the tiger mosquito *Aedes albopictus* is extending its distribution range worldwide, and it has already invaded large parts of the Mediterranean [1].

## Dengue and chikungunya becoming endemic in Europe?

Since 2010, sporadic cases of locally acquired dengue have been notified in Europe and an outbreak occurred on Madeira Island between the week 39 of 2012 and the week 9 of 2013. Important drivers of these events are viraemic travellers and the invasion of both vector mosquito species *Ae*. *aegypti* and *Ae*. *albopictus* [1]. Recently, new autochthonous dengue cases were reported in southern France in 2014 [3,4] and 2015 [5], demonstrating the vulnerability of Europe to dengue.

**Fig 1:**
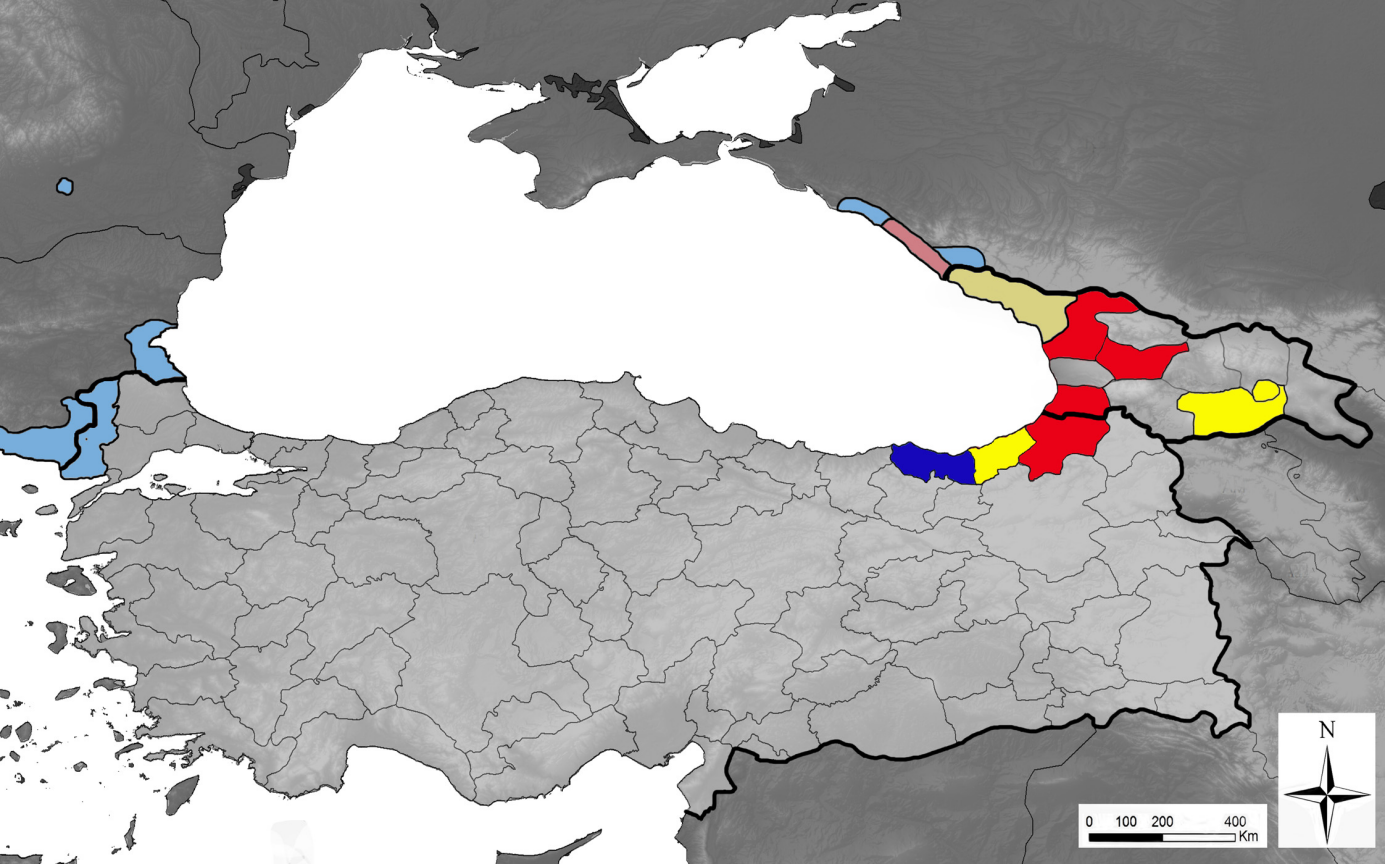
Current known distribution of *Aedes aegypti* and *Aedes albopictus* in the Black Sea region. Presence of the mosquito species is shown at province/district level (except for Russia, where the colonised area is much undersized). Light colours: known distribution up to August 2015; Dark colours: surveillance results, September 2015; Yellow: presence of *Aedes aegypti*, the yellow fever mosquito, only; blue: presence of *Aedes albopictus*, the tiger mosquito, only; red, presence of both *Ae*. *aegypti* and *Ae*. *albopictus*. AM: Armenia; AZ: Azerbaijan; BG: Bulgaria; GE: Georgia; GR: Greece; IQ: Iraq; IR: Iran; RO: Romania; RU: Russia; SY: Syria; TR: Turkey; UA: Ukraine.

Therefore it is crucial to extend and strengthen surveillance of the invasive *Aedes* mosquitoes and to address the need for the rapid suppression if not elimination of newly introduced *Ae*. *aegypti* populations in the European region. This is of particular importance in southern Europe and the Caucasus, where *Ae*. *aegypti* was historically present. Recently the European Centre for Disease Prevention and Control (ECDC) and the European Food Safety Agency (EFSA) have initiated the VectorNet scheme (as an extension of Vbornet scheme), a network that aims to support these agencies in their preparedness for vector-borne diseases in the framework of the One-Health concept. The network, among others, gathers distribution data of major arthropod vectors. Information collected from the Black Sea region has already revealed the presence of *Aedes albopictus* in western Turkey (Edirne province, bordering Greece), Bulgaria, Romania, southern Russia (Sochi region) and Abkhazia, as well as the occurrence of *Ae*. *aegypti* in these two last territories [1, 6]. In order to complete our knowledge on the geographical spread of these species, we have performed field work in September 2015 to collect data on the distribution of invasive *Aedes* mosquitoes in Georgia and north-eastern Turkey. Significant findings of these studies have been (1) the presence of both *Ae*. *aegypti* and *Ae*. *albopictus* over extended areas of Georgia including *Ae*. *aegypti* in the capital city Tbilisi, and (2) the spread of both species into north-eastern Turkey (Fig 1). Adult populations of these two invasive mosquitoes showed being anthropophagic and were found at several locations (e.g. Batumi and coastal Black Sea localities). Immature mosquito aquatic stages were found in particular in used tyres stored in outdoor conditions. Specimens were identified by morphology and some confirmed by molecular methods. These original observations are suggestive of a high probability of further spread of both invasive mosquito species in particular to ports of the Black Sea via ships and ferries, and via ground vehicles to places frequented by tourists and into major cities of Turkey including Istanbul. This might be the presages of re-colonisation of Mediterranean Europe by *Ae*. *aegypti*.

Currently, infectious diseases caused by viruses transmitted by both *Ae*. *aegypti* and *Ae*. *albopictus* are a growing global health concern. Dengue has shown a 30-fold increase in global incidence during the past 50 years, affecting more than 100 countries throughout tropical and subtropical regions of the world [7]. Chikungunya was restricted to limited parts of Africa and Asia until 2005, when it spread for the first time to territories of the Indian Ocean, but is now occurring globally [8]. Also Zika virus has recently become a global player, after its emerging in the Pacific [9], and now in the Americas [10].

In a number of western European countries [1] preparedness plans including surveillance of invasive mosquito vectors, of which results are gathered quarterly on VectorNet maps [11], and in some cases regional or national integrated plans combining surveillance and control of both vectors and diseases are implemented or under development (e.g. France, Italy, Belgium, Switzerland). Surveillance of invasive vectors (presence, spread, activity, and abundance) and detection of dengue and chikungunya cases (both introduced and autochthonous) aim at collecting data to estimate the risk level. Furthermore, there are also prevention plans in place that categorise stakeholders, define information flows, and list measures that may be activated according to the faced risk level. These might include application of focal vector control measures around disease cases and in areas where a competent vector is established and active, and complementary measures such as informing the public about mosquito bite prevention. Such integrated plans support preparedness and allow rapid implementation of adapted responses. It is now crucial to rapidly define such plans in all countries where *Ae*. *aegypti* or *Ae*. *albopictus* are established. Local physicians’ capacities to rapidly identify and notify these arboviral infections should be enhanced. International collaboration, in particular around the Black Sea, is crucial in order to support local capacities and boost Europe’s preparedness, which is essential for planning adequate and efficient measures in both pre-emptive situations as well as in outbreak response (Box 1). WHO member states already agreed on an international strategy for surveillance and control of invasive mosquito vectors and re-emerging vector-borne diseases [12].

**Box 1. Improve preparedness for dengue, chikungunya, and Zika infection in Europe.**

- Public health authorities, physicians, and scientists should familiarise themselves with dengue, chikungunya, and Zika infections and prepare appropriately.
- As long as no dengue/chikungunya/Zika-specific prophylaxis or therapeutics are available, sustainable vector management is the only currently available approach for prevention and control.
- ECDC guidelines for the surveillance of mosquito vectors [13,14], can be applied to guide mosquito surveillance plans
- Integrated surveillance and control programmes should be generalised, at least in the Mediterranean and the Black Sea regions.

## Eliminating *Aedes aegypti* from Europe?

It has been observed that the elimination of an invasive mosquito species such as *Ae*. *albopictus* is extremely difficult if not impossible [15,16]. Although elimination could be achieved during the last century at some places in Europe and the Americas for *Ae*. *aegypti* [1,17], recent eradication plans have failed in most cases (e.g. USA) [18,19]. In Europe, *Ae*. *aegypti* was successfully eliminated following its introduction into the Netherlands [20], but it is doubtful that the species would have managed to successfully overwinter under the local climatic conditions anyway. This is in contrast however to the situation in the Portuguese Autonomous Region of Madeira, where *Ae*. *aegypti* maintains its presence, with some signs of expansion throughout the Island of Madeira despite active control campaigns implemented in particular during the large dengue outbreak in 2012–13 [21]. Major challenges include the difficulties in implementing control measures on privately owned land, and the limited effectiveness of classical control methods [22]. Thus, novel methods are needed to complete the panel of measures for sustainable integrated vector management. At least, control measures aiming at slowing down the rate of mosquito spread and/or suppress the mosquito population during periods of elevated risk of pathogen transmission should be implemented rapidly. Otherwise, assuming that pathogens are imported via travellers, outbreaks of dengue or chikungunya might become more frequent and Zika could emerge in Europe.
