# Correction: Spread of the Invasive Mosquitoes *Aedes aegypti* and *Aedes albopictus* in the Black Sea Region Increases Risk of Chikungunya, Dengue, and Zika Outbreaks in Europe

**DOI:** 10.1371/journal.pntd.0004764

**Published:** 2016-05-27

**Authors:** Muhammet M. Akiner, Berna Demirci, Giorgi Babuadze, Vincent Robert, Francis Schaffner

The text in Fig 1 is missing. Please view the correct Fig 1 here.

**Fig 1:**
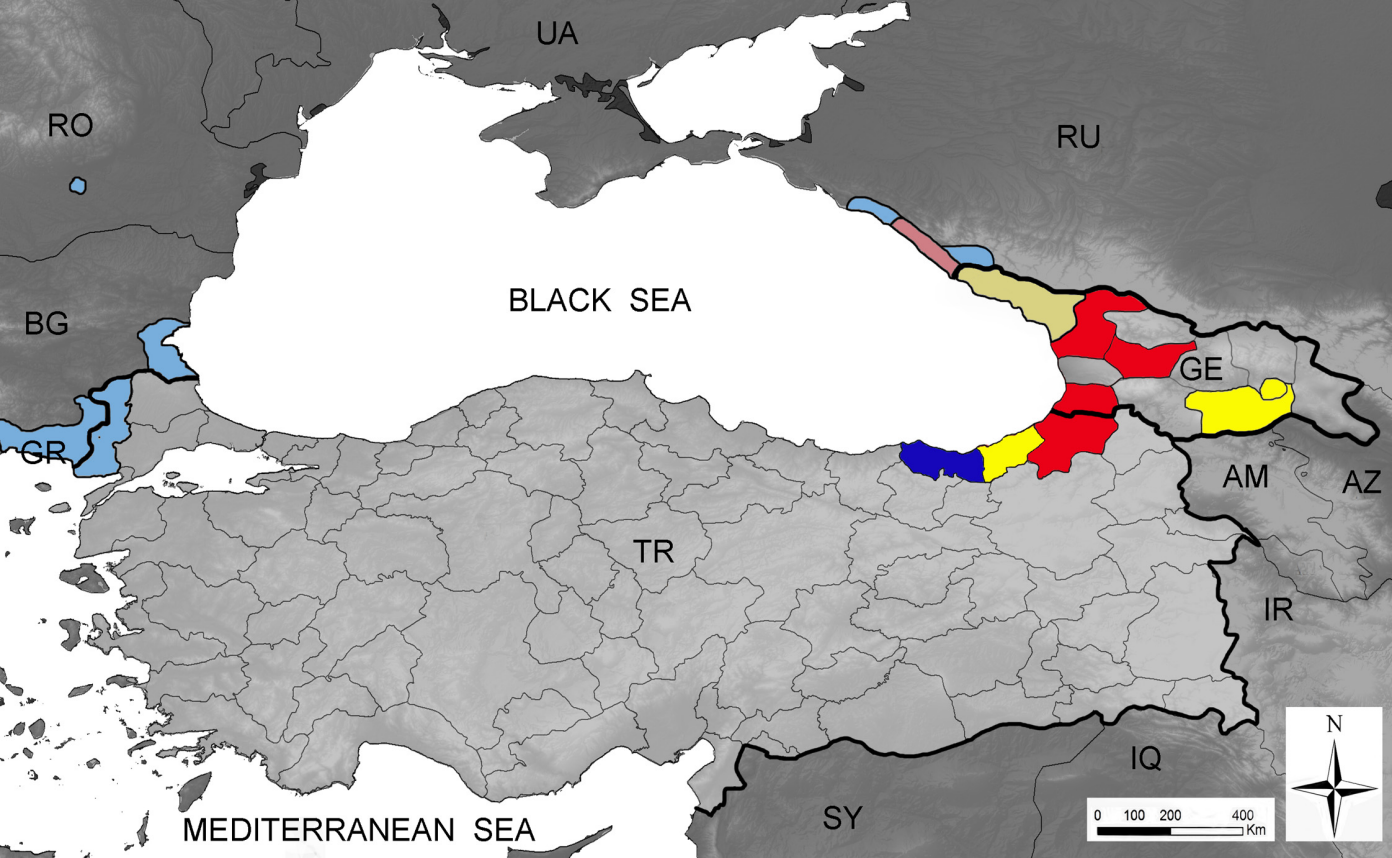
Current known distribution of Aedes aegypti and Aedes albopictus in the Black Sea region. Presence of the mosquito species is shown at province/district level (except for Russia, where the colonised area is much undersized). Light colours: known distribution up to August 2015; Dark colours: surveillance results, September 2015; Yellow: presence of Aedes aegypti, the yellow fever mosquito, only; blue: presence of Aedes albopictus, the tiger mosquito, only; red, presence of both Ae. aegypti and Ae. albopictus. AM: Armenia; AZ: Azerbaijan; BG: Bulgaria; GE: Georgia; GR: Greece; IQ: Iraq; IR: Iran; RO: Romania; RU: Russia; SY: Syria; TR: Turkey; UA: Ukraine.
